# Wider sampling reveals a non-sister relationship for geographically contiguous lineages of a marine mussel

**DOI:** 10.1002/ece3.1033

**Published:** 2014-04-25

**Authors:** Regina L Cunha, Katy R Nicastro, Joana Costa, Christopher D McQuaid, Ester A Serrão, Gerardo I Zardi

**Affiliations:** 1 Centre of Marine Sciences – CCMAR, Campus de Gambelas, Universidade do Algarve8005-139, Faro, Portugal; 2 Department of Zoology and Entomology, Rhodes UniversityGrahamstown, 6140, South Africa

**Keywords:** Independent origin, *Perna perna*, phylogeographic patterns, wider sampling

## Abstract

The accuracy of phylogenetic inference can be significantly improved by the addition of more taxa and by increasing the spatial coverage of sampling. In previous studies, the brown mussel *Perna perna* showed a sister–lineage relationship between eastern and western individuals contiguously distributed along the South African coastline. We used mitochondrial (COI) and nuclear (ITS) sequence data to further analyze phylogeographic patterns within *P. perna*. Significant expansion of the geographical coverage revealed an unexpected pattern. The western South African lineage shared the most recent common ancestor (MRCA) with specimens from Angola, Venezuela, and Namibia, whereas eastern South African specimens and Mozambique grouped together, indicating a non-sister relationship for the two South African lineages. Two plausible biogeographic scenarios to explain their origin were both supported by the hypotheses-testing analysis. One includes an Indo-Pacific origin for *P. perna*, dispersal into the Mediterranean and Atlantic through the Tethys seaway, followed by recent secondary contact after southward expansion of the western and eastern South African lineages. The other scenario (Out of South Africa) suggests an ancient vicariant divergence of the two lineages followed by their northward expansion. Nevertheless, the “Out of South Africa” hypothesis would require a more ancient divergence between the two lineages. Instead, our estimates indicated that they diverged very recently (310 kyr), providing a better support for an Indo-Pacific origin of the two South African lineages. The arrival of the MRCA of *P. perna* in Brazil was estimated at 10 [0–40] kyr. Thus, the hypothesis of a recent introduction in Brazil through hull fouling in wooden vessels involved in the transatlantic itineraries of the slave trade did not receive strong support, but given the range for this estimate, it could not be discarded. Wider geographic sampling of marine organisms shows that lineages with contiguous distributions need not share a common ancestry.

## Introduction

Increased taxon sampling recognizably improves phylogenetic inference (Hillis 1996) while in ecology, the understanding of distribution patterns depends critically on the scales at which observations are made (Wiens 1989; Rahbek 2005). The analysis of a small subset of species that are presumed to be representative can distort phylogenetic relationships (Tuinen et al. 2000; Murphy et al. 2001). This can be mitigated by large sequence data sets that improve the accuracy of phylogenetic estimation (Poe and Swofford 1999; Heath et al. 2008), but using only a few representatives from a particular group can produce misleading results and incorrect topologies even with increased sequence length (Zwickl and Hillis 2002; Heath et al. 2008).

Here, we evaluated the effect of increasing the scale of geographic sampling on the reconstruction of phylogenetic and phylogeographic patterns. As a model system, we used marine mussels of the genus *Perna*, focusing on the South African coastline, where three main biogeographic regions exist across a wide range of climatic and oceanographic conditions (Harrison 2002). These regions have been defined by the rocky shore biota and include a cool-temperate west coast, a warm-temperate south coast, and a subtropical east coast (Stephenson and Stephenson 1972; Emanuel et al. 1992). The genus *Perna* comprises three currently recognized species of intertidal mussels: *P. perna, P. canaliculus*, and *P. viridis*. A previous phylogenetic study of the genus based on the mitochondrial cytochrome oxidase subunit I (COI) gene and the internal transcribed spacers of the nuclear ribosomal RNA coding region (ITS) showed that a fourth putative species, *P. picta*, clustered within the *P. perna* clade and thus, was not a valid taxon (Wood et al. 2007).

**Figure 1:**
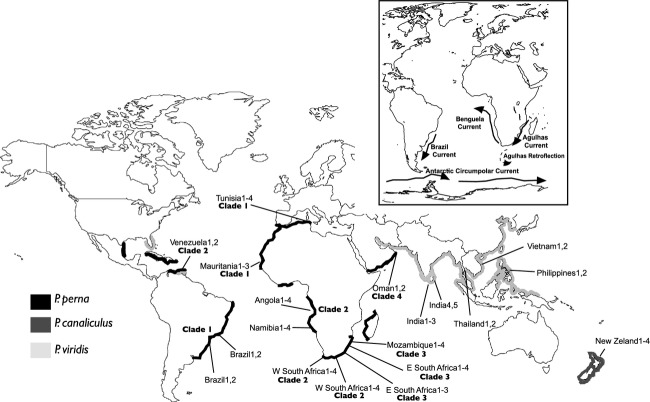
Geographical distribution and sampling locations of the species within the genus *Perna (P. viridis, P. perna* and *P. canaliculus*) used in this study***.*** Sampling location codes are further explained in Table 1. The inset shows the main oceanic currents in the studied area.

*Perna perna* Linnaeus, 1758 has a wide distribution, occurring in warm-temperate regions of the Atlantic, Mediterranean Sea, and Indian Ocean, including the western and east coasts of South Africa, and has recently become invasive in the Gulf of Mexico ((Hicks and Tunnell 1993); Fig. 1). The absence of fossil records of *P. perna* in Brazil suggests a recent introduction in this area (Silva and Barros 2011), and it has been claimed that the presence of brown mussels in Brazil could have resulted from the hull fouling in wooden vessels involved in the transatlantic itineraries of the slave trade (Souza et al. 2008), the so-called “Slave Route” (Harris 2006).

Phylogeographic studies focusing on South African coastal species (e.g., estuarine crustaceans (Teske et al. 2008, 2006), limpets (Ridgway et al. 2006), and intertidal mussels (Zardi et al. 2007, 2011)) have identified remarkable barriers to gene flow located at the borders of these biogeographic regions. Overall, the reconstructed phylogeographic patterns have included either cold-water (west) versus warm/subtropical (southeastern) or warm-temperate (south) versus subtropical (east) sister–lineage relationships (Ridgway et al. 2006; Teske et al. 2006, 2008). In South African *P. perna*, a western and an eastern lineages were identified (Zardi et al. 2007), suggesting that two sister lineages have diverged in response to sharp ecological/oceanographic barriers.

Phylogenetic and phylogeographic studies of *P. perna* have thus far comprised a smaller coverage of its geographic distribution, including either only specimens from Brazil, Venezuela, and the east coast of South Africa (Wood et al. 2007), or South African and Namibian specimens (Zardi et al. 2007). We expanded intraspecific sampling to evaluate whether the addition of specimens from almost the entire range of *P. perna*, and the inclusion of congeneric species, would affect interpretation of the phylogeography of the species, particularly the questions that remain unanswered regarding its colonization across the Southern Atlantic. We used the same molecular markers (COI and ITS) as Wood et al. (2007) to allow comparison. We also performed a Bayesian molecular clock dating analysis using two independent partitions (COI and ITS) and unlinked evolutionary models as implemented in Beast (Drummond et al. 2012) to date lineage-splitting events within the genus *Perna*.

Our goals were: (1) to assess the impact of increasing taxon and geographic extent of sampling on our perception of the genetic boundaries of a marine organism; (2) to date the main lineage-splitting events within the genus *Perna*, particularly focusing on the origin of the two South African lineages of *P. perna*, and (3) to test alternative hypotheses and establish a phylogeographic scenario for the origins of the two South African lineages.

## Materials and Methods

### Specimen collection, DNA extraction, amplification, and sequencing

A total of 31 specimens of the brown mussel *P. perna* were collected from eight different locations that included the Mediterranean, Gulf of Oman, Angola, Namibia, Mozambique, and South Africa (see Table 1 for further details). All specimens were preserved in 96% ethanol. Sequences of other species within the genus *Perna (P. viridis* and *P. canaliculus*) were retrieved from the GenBank (Table 1).

**Table 1:** List of species used in this study, sample location, and GenBank accession numbers. In bold, sequences from this study

			GenBank accession no.	
			
Species	Code	Location	COI	ITS
*Perna perna*	Tunisia1	Tunisia – Bizerte	**KC691986**	**KC692016**
*Perna perna*	Tunisia2	Tunisia – Bizerte	**KC691987**	**KC692017**
*Perna perna*	Tunisia3	Tunisia – Bizerte	**KC691988**	**KC692018**
*Perna perna*	Tunisia4	Tunisia – Bizerte	**KC691989**	**–**
*Perna perna*	W South Africa1	South Africa – Gans Bay	**KC691990**	**KC692019**
*Perna perna*	W South Africa2	South Africa Gans Bay	**KC691991**	**KC692020**
*Perna perna*	W South Africa3	South Africa Gans Bay	**KC691992**	**–**
*Perna perna*	W South Africa4	South Africa Gans Bay	**KC691993**	**–**
*Perna perna*	W South Africa5	South Africa Plettenberg	**KC691994**	**KC692021**
*Perna perna*	W South Africa6	South Africa Plettenberg	**KC691995**	**KC692022**
*Perna perna*	W South Africa7	South Africa Plettenberg	**KC691996**	**KC692023**
*Perna perna*	W South Africa8	South Africa Plettenberg	**KC691997**	**KC692024**
*Perna perna*	E South Africa1	South Africa Umhlanga	**KC691998**	**KC692025**
*Perna perna*	E South Africa2	South Africa Umhlanga	**KC691999**	**KC692026**
*Perna perna*	E South Africa3	South Africa Umhlanga	**KC692000**	**KC692027**
*Perna perna*	E South Africa4	South Africa Umhlanga	**–**	**KC692028**
*Perna perna*	E South Africa5	Eastern South Africa	DQ917618	DQ924559
*Perna perna*	E South Africa6	Eastern South Africa	DQ917617	**–**
*Perna perna*	E South Africa7	Eastern South Africa	DQ917616	**–**
*Perna perna*	Angola1	Angola – Luanda	**KC692001**	**KC692029**
*Perna perna*	Angola2	Angola – Luanda	**KC692002**	**KC692030**
*Perna perna*	Angola3	Angola – Luanda	**KC692003**	**–**
*Perna perna*	Angola4	Angola – Luanda	**KC692004**	**–**
*Perna perna*	Namibia1	Namibia – Swakopmund	**KC692005**	**KC692031**
*Perna perna*	Namibia2	Namibia – Swakopmund	**KC692006**	**KC692032**
*Perna perna*	Namibia3	Namibia – Swakopmund	**KC692007**	**–**
*Perna perna*	Namibia4	Namibia – Swakopmund	**KC692008**	**–**
*Perna perna*	Mozambique1	Mozambique – Punta D'Ouro	**KC692009**	**KC692033**
*Perna perna*	Mozambique2	Mozambique – Punta D'Ouro	**KC692010**	**KC692034**
*Perna perna*	Mozambique3	Mozambique – Punta D'Ouro	**KC692011**	**KC692035**
*Perna perna*	Mozambique4	Mozambique – Punta D'Ouro	**KC692012**	**KC692036**
*Perna perna*	Oman1	Oman – Muscat	**KC692013**	**KC692037**
*Perna perna*	Oman2	Oman – Muscat	**KC692014**	**KC692038**
*Perna perna*	Oman3	Oman – Muscat	**KC692015**	**–**
*Perna perna*	Venezuela1	Venezuela	DQ917588	DQ924543
*Perna perna*	Venezuela2	Venezuela	DQ917587	DQ924542
*Perna perna*	Brazil1	Brazil – Santa Catarina	DQ917594	DQ924547
*Perna perna*	Brazil2	Brazil – Santa Catarina	DQ917593	**–**
*Perna perna*	Brazil3	Brazil – São Paulo	DQ917592	DQ924546
*Perna perna*	Brazil4	Brazil – São Paulo	DQ917591	DQ924545
*Perna perna (*former *P. pict*a)	Morocco1	Morocco	DQ917603	**–**
*Perna perna (*former *P. pict*a)	Morocco2	Morocco	DQ917602	**–**
*Perna perna (*former *P. pict*a)	Morocco3	Morocco	DQ917601	**–**
*Perna perna (*former *P. pict*a)	Morocco4	Morocco	DQ91760	**–**
*Perna perna (*former *P. pict*a)	Mauritania1	Mauritania	DQ917597	DQ924548
*Perna perna (*former *P. pict*a)	Mauritania2	Mauritania	DQ917596	**–**
*Perna perna (*former *P. pict*a)	Mauritania3	Mauritania	DQ917595	**–**
*Perna canaliculus*	New Zealand1	New Zealand	DQ917607	DQ924551
*Perna canaliculus*	New Zealand2	New Zealand	DQ917613	DQ924556
*Perna canaliculus*	New Zealand3	New Zealand	DQ917608	DQ924552
*Perna canaliculus*	New Zealand4	New Zealand	DQ917609	DQ924553
*Perna viridis*	India1	India	DQ917612	DQ924555
*Perna viridis*	India2	India	DQ917611	DQ924554
*Perna viridis*	India3	India	DQ917610	**–**
*Perna viridis*	India4	Southern India	DQ917586	DQ924541
*Perna viridis*	India5	Southern India	DQ917585	DQ924540
*Perna viridis*	Philippines1	Philippines	DQ917599	DQ924550
*Perna viridis*	Philippines2	Philippines	DQ917598	DQ924549
*Perna viridis*	Thailand1	Thailand	DQ917590	DQ924544
*Perna viridis*	Thailand2	Thailand	DQ917589	**–**
*Perna viridis*	Vietnam1	Vietnam	DQ917584	DQ924539
*Perna viridis*	Vietnam2	Vietnam	DQ917583	DQ924538
*Aulacomya atra maoriana*	**–**	New Zealand	DQ917614	DQ924558
*Mytilus galloprovincialis*	**–**	New Zealand	DQ917605	**–**
*Mytilus edulis*	**–**	UK – Wales	DQ917606	AY695798
*Modiolus areolatus*	**–**	New Zealand	DQ917604	**–**
*Xenostrobus pulex*	**–**	New Zealand	DQ917582	**–**

Total genomic DNA was isolated from muscle tissue (foot) using the cetyltrimethylammonium bromide (CTAB) protocol (Doyle and Doyle 1987). Universal primers from Folmer et al. (1994) were used to amplify a portion (approximately 650 bp) of the mitochondrial COI gene. In addition, a partial fragment of ribosomal DNA spanning 18S, internal transcribed spacer 1 (ITS1), 5.8S, ITS2, and 28S rRNA was amplified using primers ITS5 (White et al. 1990) and ITS28 cc of Wagstaff and Garnock-Jones 1998. Amplification protocols for the ITS and COI fragments are described in Wood et al. 2007 and Zardi et al. 2007, respectively. Sequences were deposited in GeneBank under the accession numbers given in Table 1.

### Sequence alignment and phylogenetic analyses

DNA sequences were aligned with Mafft v5 (Katoh and Toh 2010) using the auto option that automatically selects the appropriate strategy according to data size. To perform phylogenetic analyses, three different data sets were analyzed: (1) partial nucleotide sequences of the mitochondrial COI of 66 specimens representing three species of the genus *Perna* (*P. perna*, *P. viridis*, and *P. canaliculus*) and five out-groups (*Mytilus edulis*, *M. galloprovincialis*, *Aulacomya atra maoriana*, *Modiolus areolatus*, and *Xenostrobus pulex* – GenBank accession numbers in Table 1) produced an alignment of 623 base pairs (bp); (2) nuclear ITS sequences of 45 mussels representing the three *Perna* species and the out-group *A. atra maoriana* produced an alignment of 865 bp, and (3) a concatenated data set with partial nucleotide sequences of the mitochondrial COI and the nuclear sequences of 42 mussels representing the three *Perna* species and the outgroup *Mytilus edulis* produced an alignment of 1580 bp. The selection of out-group taxa was based on previous work by Wood et al. (2007).

The Akaike information criterion (AIC) (Akaike 1973) implemented in Modeltest v.3.7 (Posada and Crandall 1998) was used to determine the evolutionary models that best fitted the data sets. Bayesian inferences (BI) based on the three data sets were conducted with MrBayes v3.2.1 (Ronquist et al. 2012). Four Metropolis-coupled Markov chain Monte Carlo (MCMC) analyses were run for two million generations, and sampled every 100 generations. Two independent runs were performed for each data set. The three data sets were analyzed under the GTR+Γ, the best-fit model selected by Modeltest. The burn-in was set to 120,000 generations for the mitochondrial and nuclear data sets, and to 100,000 steps for the concatenated data set. Robustness of the inferred trees was evaluated using Bayesian posterior probabilities (BPPs). Maximum likelihood (ML) analyses were performed with PhyML v2.4.4 (Guindon and Gascuel 2003) using the three data sets and parameters obtained with Modeltest. The robustness of the inferred trees was tested by nonparametric bootstrapping (BP) using 1000 pseudoreplicates.

### Testing alternative biogeographic scenarios

Approximately unbiased (AU) (Shimodaira 2002), the Kishino–Hasegawa (KH) (Kishino and Hasegawa 1989), and the Shimodaira–Hasegawa (SH) (Shimodaira and Hasegawa 1999) tests were used to evaluate different topologies representing alternative scenarios (see the discussion for further details on the alternative biogeographic scenarios) for the origin of *P. perna* in South Africa based on the ML topology obtained with the combined data set (42 taxa, 1488 bp). The null hypothesis is that the ML topology is identical to the alternative scenarios. Tests were performed using PaML (Yang 2007) and Consel (Shimodaira and Hasegawa 2001).

### Dating analysis and species tree inference

We used a Bayesian relaxed molecular clock approach as implemented in Beast v1.7.4 (Drummond et al. 2012) to date lineage-splitting events within the genus *Perna*. We used a Yule tree prior that assumes a constant speciation rate among lineages. This method allows the incorporation of fossil uncertainties because it uses probabilistic calibration priors instead of point calibrations (Drummond et al. 2006).

Mitochondrial COI (16 taxa, 617 bp) and nuclear ITS (16 taxa, 963 bp) data sets were treated as two independent loci with independent substitution models as selected by Modeltest (COI: TN93+Γ; ITS: GTR+Γ), but sharing a single tree partition. Two calibration points were provided by placing a lognormal prior distribution on the age estimate for the divergence between *Perna* and *Mytilus* (mean in real space: 0.47, log standard deviation = 0.423 and Offset = 37.2; interval: 40.4–37.2 myr) and between *P. canaliculus* and *P. perna* (mean in real space: 0.3, log standard deviation = 0.422 and Offset = 2.6; interval: 5.3–2.6 myr). The first calibration point was based on the approximate age of a fossil found in the Antarctic Peninsula, Seymour Island from the Lower Eocene [40.4–37.2] myr assigned to the genus *Perna* (Stilwell and Zinsmeister 1992). The second calibration was based on a fossil of *P. canaliculus* from the Pliocene [5.3–2.6] myr from New Zealand (Beu 2004).

MCMC analyses were run for 900,000,000 generations with a sample frequency of 10,000, following a discarded burn-in of 9,000,000 steps. The convergence to the stationary distributions was confirmed by inspection of the MCMC samples using Tracer v1.5 (Rambaut et al. 2013).

All dating analyses were performed on the CCmar Computational Cluster Facility (http://gyra.ualg.pt) at the University of Algarve.

## Results

### Phylogenetic relationships of *Perna* mussels

**Figure 2:**
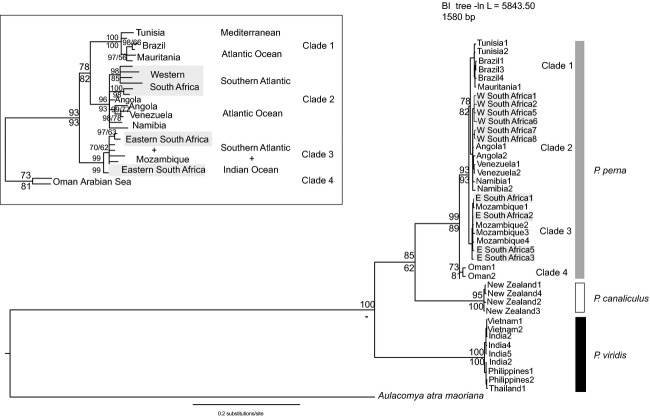
Phylogenetic relationships of the currently recognized species within the genus *Perna*. The Bayesian topology based on a combined data set (mitochondrial COI and nuclear ITS) is shown. Numbers above and below nodes are ML bootstrap values and Bayesian posterior probabilities, respectively. Gray boxes indicate western and eastern South African lineages. The inset shows phylogeographic patterns of diversification within *P. perna*.

Potential scale reduction factors in Bayesian analyses (all data sets) were about 1.00, indicating convergence of the runs (Gelman and Rubin 1992). BI and ML analyses based on the concatenated dataset recovered identical topologies with the single exception that the relative phylogenetic position of *P. viridis* as the sister species to the remaining *Perna* received no statistical support in the latter. BI analysis based on the concatenated dataset recovered three well-supported clades within *P. perna* (Fig. 2), whereas only a single clade was recovered in the nuclear-based BI analyses (Material S2). The three clades included specimens from: (1) Tunisia, Morocco, Mauritania, and Brazil; (2) Venezuela, western South Africa, and Namibia, and (3) eastern South Africa and Mozambique. Specimens from Oman were recovered as the sister group of the remaining *P. perna* (Fig. 2).

In the ML analyses based both on COI (Material S1) and nuclear (Material S2) data sets, the relative phylogenetic position of Oman individuals remained unresolved.

### Testing alternative biogeographic scenarios

**Figure 3:**
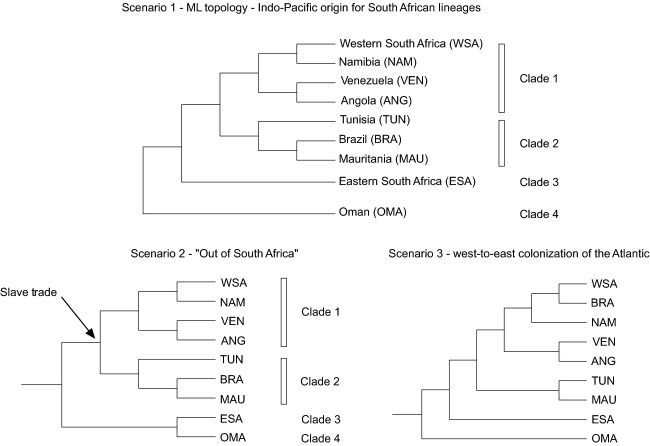
Alternative biogeographic scenarios for the origins of the two South African *Perna perna* lineages.

Results of AU, SH, and KH tests of alternative tree topologies are summarized in Table 2. All three tests rejected the alternative scenario that indicates a west-to-east colonization of the Atlantic, whereas the “Out of South Africa” (Fig. 3) hypothesis could not be rejected by any of the tests (*P* > 0.05).

**Table 2:** Log-likelihood and *P* values of Approximately Unbiased (AU), Shimodaira–Hasegawa (SH), and Kishino–Hasegawa (KH) tests for each of the alternative biogeographic scenarios. The first topology corresponds to the optimal ML tree based on the concatenated data set. Topologies are rejected at the 5% significance level

Scenarios	Alternative topologies	−log L	AU	SH	KH
ML tree	(((clade1, clade2), clade3), clade4);	5885.8	0.944	0.973	0.929
“Out of South Africa”	((clade1, clade2), (clade3, clade4));	5891.5	5.60E-02	4.20E-01	7.10E-02
West-to-east colonization	(((((((WSA,BRA),NAM),(VEN,ANG)),(TUN,MAU)),ESA),OMA));	5970.3	1.00E-06	4.00E-05	4.00E-05

ANG, Angola; BRA, Brazil; ESA, eastern South Africa; MAU, Mauritania; NAM, Namibia; OMA, Oman; TUN, Tunisia; VEN, Venezuela; WSA, western South Africa.

### Dating analyses

The ultrametric tree obtained from the Beast analysis showed effective sample size values >200, which indicates convergence of the runs. Age estimates showed narrow confidence intervals, which reflects the accuracy of the analysis.

**Figure 4:**
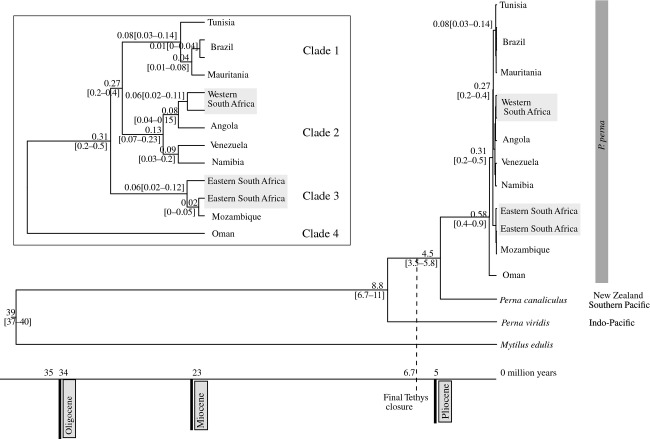
Maximum clade credibility chronogram obtained with Beast showing divergence dates of main splitting events within the genus *Perna*. Age estimates were based on two partitions of the data set (mitochondrial COI and nuclear ITS) with independent evolutionary models (COI: TN93+Γ; ITS: GTR+Γ). Gray boxes indicate western and eastern South African lineages. The inset shows age estimates (in million years) of the splitting events within *P. perna*.

*Perna viridis* diverged from the remaining *Perna* species between 8.8 myr ago, and *P. canaliculus* at 4.5 myr (Fig. 4). The age of the most recent ancestor common to both South African lineages was estimated at 310 [200–500] kyr. The origin of the eastern and western South African lineages was estimated at 60 kyr. The age of the MRCA of the Brazilian lineage of *P. perna* was estimated at 10 [0–40] kyr.

## Discussion

The novel geographical coverage analyzed in this paper revealed a complex evolutionary history of *Perna perna* that would have been incorrectly inferred by analyses at regional scales that miss the global evolutionary history. The results suggest a non-sister relationship for the two South African lineages and anthropogenic introduction in Central and South America through the slave trade routes, resulting in a complex spatial mosaic of populations.

### Effect of increasing geographic sampling on the reconstructed phylogenetic patterns

The most significant finding from our study is the existence of an independent origin for the eastern and western South African lineages of *P. perna* as they were not recovered as sister groups in any of the analyses performed here. Thus, setting these two lineages in a larger taxonomic and geographic context has completely altered our understanding of their evolutionary history.

Despite the relatively long larval period of the brown mussel *P. perna* (assumed to be similar to that of *Mytilus edulis* at 15–20 days with a maximum of 40 days, (Bayne 1975)), a strong phylogeographic break has been observed on the South African coastline, and interpreted as indicating a sister–lineage relationship between eastern and western populations (Zardi et al. 2007). Similar genetic discontinuities in other invertebrates have been found within this region, again interpreted as sister–lineage relationships (e.g., (Teske et al. 2007, 2006). Such patterns suggested differentiation across a sharp environmental barrier, but are also compatible with the alternative hypothesis that this area represents a contact zone between independently evolved lineages.

Using a wider context, our reconstructed major clades included a sister relationship between specimens from Tunisia, Mauritania, and Brazil (Clade 1) and western South Africa, Angola, Namibia, and Venezuela (Clade 2). Clade 3 was composed of specimens from eastern South Africa grouping together with Mozambique (Fig. 2). Given the geographical proximity of the two South African lineages, and previous results that showed a sister–lineage relationship between them (Zardi et al. 2007), this is a completely unexpected pattern. Thus far, all studies involving South African phylogeographic breaks either comprised representatives exclusively from this region (Grant et al. 1992; Ridgway et al. 2006; Teske et al. 2006, 2007) or also included specimens from Namibia (Zardi et al. 2007). The wider coverage of the geographic distribution of *P. perna* presented here revealed a very different phylogeographic pattern for the species. Further studies are required to determine whether extending the geographic coverage of sampling would yield similar results for other marine organisms.

### Slave trade routes and present-day distribution of *P. perna*

The transatlantic slave trade started in the 15th century and lasted more than 500 years. More than 30 million people were exchanged between Africa and three continents (North and South America, and Europe). Slave trade routes during the 15th and 16th centuries were predominantly from northwestern Africa (Senegal and Gambia) to the Caribbean and Brazil (Beckles 2002; Harris 2006). In the following centuries (17th, 18th, and 19th), routes moved toward the South with intense slave trade between central west Africa (Angola, Equatorial Guinea, and Congo), Brazil and the Caribbean (Beckles 2002; Harris 2006).

If the introduction of *P. perna* to Brazil and the Caribbean (Venezuela) is to be explained by the “Slave Trade Route” hypothesis (Souza et al. 2008), based on the above itineraries, colonization of this area could have been either from northwestern Africa (Senegal and Gambia) or from central west Africa (Angola). To establish the following colonization pathways, some assumptions were made. Given the vicinity to Senegal, where most of the departures to the Caribbean region occurred, we assumed that *P. perna* from Mauritania could also have colonized the Caribbean (Venezuela). The same assumption is valid for Namibia due to its proximity to Angola.

The colonization of Brazil from northwestern Africa is expected to generate a topology in which specimens from Brazil and Mauritania group together. Alternatively, if the colonization occurred from central west Africa, this is the expected phylogenetic tree: (Brazil, (Angola, Namibia)). If colonization of the Caribbean (Venezuela) was from northwestern Africa or from the central west Africa, the two possible topologies are: (Venezuela, Mauritania) or (Venezuela, (Angola, Namibia)), respectively.

Our phylogenies (Figs. 2 and 4) always revealed two clades: the first included Brazil and Mauritania (Clade 1); the second clade included Venezuela, Angola, Namibia, and western South Africa (Clade 2). Thus, our results indicate colonization of Brazil from Mauritania, which is consistent with one of the alternative scenarios (northwestern Africa – Mauritania vs. central west Africa – Angola). Our dating analyses (Fig. 4) estimated the origin of the MRCA of the Brazilian *P. perna* populations at about 10 kyr. This seems to contradict the hypothesis of a relatively recent (±500 years) introduction to Brazil. However, the confidence interval for this estimate was between 0 and 40 kyr, which encompasses the last 500 years. Given the different topologies obtained with Bayesian dating estimates or with BI analyses, the colonization of Venezuela remains uncertain.

There has been an increasing controversy regarding the estimation of mutation rates in recently diverged populations due to the existence of “time-dependency” that can cause an overestimation of rates (Ho et al. 2005, 2011). Consequently, given their recent divergence, our estimates for the *P. perna* lineages might be increased, making the recent introduction of the brown mussel to Brazil through the slave routes more plausible.

### Biogeographic scenarios for the origin of the two South African lineages of *P. perna*

Our phylogenetic analyses suggest that the origin of the two South African lineages may be explained by more than one biogeographic scenario (Fig. 3).

Scenario (1): an Indo-Pacific origin for *P. perna*, dispersal into the Mediterranean and Atlantic through the Tethys seaway, and recent secondary contact after southward expansion of the western and eastern South African lineages.
Scenario (2), “Out of South Africa”: The occurrence of an historical vicariance event separated the two South African lineages, which then expanded northward into the Atlantic and Indo-Pacific.
Scenario (3): a west-to-east colonization of the Atlantic from the southern Indo-Pacific around Antarctica via the Antarctic Circumpolar Current (ACC) to South America, followed by expansion out of Brazil into southwestern Africa and the Mediterranean.

The rooted phylogeny from Figure 2 is consistent with an Indo-Pacific origin for *P. perna* and dispersal into the Mediterranean. The idea that the dispersal of *Perna* mussels between the Indo-Pacific and the Atlantic occurred through the Mediterranean by the Tethys seaway – scenario (1) – was previously proposed by Wood et al. (2007). According to our results, the MRCA of the genus occurred at 8.8 [6.7–11] myr (Fig. 4). This age estimate predates the final closure of the Tethys seaway, which remained intermittently open until the Late Miocene, 6.7 myr ago (Sonnenfeld 1985; Robba 1987). This would have allowed the colonization of the Atlantic through the Mediterranean before the closure of all connections.

Angolan and Namibian specimens grouped with the western South African lineage (clade 2), which is consistent with a southward expansion, but could also be interpreted as northward colonization. The simplest explanation for the presence of *Perna* in Venezuela is its introduction through the slave trade routes, as previously mentioned. The origin of the eastern South African lineage can be explained by southward dispersal from the Indian Ocean through the Agulhas Current. Our estimate for the origin of this lineage (60 kyr, Fig. 4) is consistent with the timing of the formation of this oceanographic system (5 myr ago, (Lutjeharms 2006)).

Scenario (2), in which remnants of an old vicariance event dispersed northwards into the Atlantic and the Indo-Pacific would require a sister relationship between the western and eastern South African lineages. Instead, clade 2 (western South Africa, Namibia, Angola, and Venezuela) grouped with clade 1 (Tunisia, Mauritania, and Brazil), which seems to reject this hypothesis. However, AU, SH, and KH tests indicated that the topology that supports this scenario is not significantly different from our ML topology representing the Indo-Pacific origin for the two South African lineages (Table 2 and Fig. 3) and thus, the “out of South Africa” hypothesis (scenario 2) is not rejected.

Under scenario (3), we would expect that specimens from Brazil would group with the western South African lineage, Angola, and Namibia. Instead, in both BI and ML analyses, Brazil clustered with Mauritania and Tunisia (clade 1, Figs. 2 and 4). Nevertheless, clade 1 is the sister group of clade 2 (western South Africa, Angola, and Namibia), which provides some support for a west-to-east colonization of the Atlantic via the ACC. However, the AU, KH, and SH tests clearly rejected the west-to-east colonization of the Atlantic (Table 2).

## Conclusions

Our findings are significant by showing that the inclusion of a wider geographical range of sampling and of additional taxa resulted in a radical rethinking of the relationship between coexisting lineages of our study organism. We used mitochondrial and nuclear markers in our analyses, and the sampling strategy included almost the entire geographic distribution of *P. perna*. Previous studies of the brown mussel *P. perna* showed the existence of a sister–lineage relationship between specimens from eastern and western South Africa. In contrast, the reconstructed phylogenetic patterns presented here show an unexpected pattern in which the western lineage groups with Angola, Venezuela, and Namibia and shares the MRCA with specimens from Mauritania, Brazil, and Tunisia instead of grouping with eastern South African specimens.

Two plausible biogeographic scenarios can explain the origin of the western and eastern South African lineages. One includes a recent secondary contact in South Africa after southward expansion through the Tethys seaway. The other suggests an ancient vicariant event that caused the split between the two South African lineages followed by their northward dispersal into the Atlantic and Indo-Pacific basins. With present data is not possible to distinguish between these competing hypotheses, but we would expect a more ancient divergence between the eastern and western South African lineages according to the “Out of South Africa” scenario. Instead, we obtained a very recent estimate for the divergence between the two lineages, providing better support for the Indo-Pacific origin of the two South African lineages.

The hypothesis of a recent introduction in Brazil through hull fouling in wooden vessels involved in the transatlantic itineraries of the slave trade did not receive strong support, but could not be discarded.

## Supporting Information

**Material S1.** Phylogenetic relationships of the currently recognized species within the genus *Perna*. The maximum likelihood topology of a mitochondrial data set (COI) is shown. Numbers above and below nodes correspond to ML bootstrap values and Bayesian posterior probabilities, respectively. Gray boxes indicate the three clades within *P. perna* recovered in this analysis. The inset shows phylogeographic patterns of diversification within *P. perna*.

**Material S2.** Phylogenetic relationships of the currently recognized species within the genus *Perna*. The maximum likelihood topology of a nuclear data set (ITS) using the GTR+Γ evolutionary model is shown. Numbers above and below nodes correspond to ML bootstrap values and Bayesian posterior probabilities, respectively.

## References

[b1] Akaike H, Csaksi BNPAF (1973). Information theory as an extension of the maximum likelihood principle. 2nd International symposium on information theory.

[b2] Bayne BL, Vernberg JF (1975). Reproduction in bivalve molluscs under environmental stress. Physiological ecology of estuarine organisms.

[b3] Beckles HM (2002). Slave voyages - The transatlantic trade in enslaved Africans.

[b4] Beu AG (2004). Marine Mollusca of oxygen isotope stages of the last 2 million years in New Zealand. Part 1: revised generic positions and recognition of warm-water and cool-water migrants. J. Roy. Soc. New Zeal.

[b5] Doyle JJ, Doyle JL (1987). A rapid DNA isolation for small quantities of leaf tissue. Phytochem. Bull.

[b6] Drummond AJ, Ho SYW, Philips MJ, Rambaut A (2006). Relaxed phylogenetics and dating with confidence. PLoS Biol.

[b7] Drummond AJ, Suchard MA, Xie D, Rambaut A (2012). Bayesian phylogenetics with BEAUti and the BEAST 1.7. Mol. Biol. Evol.

[b8] Emanuel B, Bustamante R, Branch G, Eekhout S, Odendaal F (1992). A zoogeographic and functional approach to the selection of marine reserves on the west coast of South Africa. S. Afr. J. Mar. Sci.

[b9] Folmer O, Black M, Hoeh W, Lutz R, Vrijenhoek R (1994). DNA primers for amplification of mitochondrial cytochrome c oxidase subunit I from diverse metazoan invertebrates. Mol. Mar. Biol. Biotech.

[b10] Gelman A, Bernardo JM, Berger J, Dawid AP, Smith AFM, Rubin D (1992). A single series from the Gibbs sampler provides a false sense of security. Bayesian Statistics 4.

[b11] Grant WS, Schneider AC, Leslie RW, Cherry MI (1992). Population genetics of the brown mussel *Perna perna* in Southern Africa. J. Exp. Mar. Biol. Ecol.

[b12] Guindon S, Gascuel O (2003). A simple, fast and accurate algorithm to estimate large phylogenies by maximum likelihood. Syst. Biol.

[b13] Harris J (2006). The Slave Route Projectin. http://www.slavevoyages.org/tast/assessment/intro-maps/01.jsp.

[b14] Harrison T (2002). Preliminary assessment of the biogeography of fishes in South African estuaries. Mar. Freshwater Res.

[b15] Heath TA, Hedtke SM, Hillis DM (2008). Taxon sampling and the accuracy of phylogenetic analyses. J. Syst. Evol.

[b16] Hicks DW, Tunnell JWJ (1993). Invasion of the South Texas Coast by the edible brown mussel *Perna perna (Linnaeus, 1758)*. Veliger.

[b17] Hillis DM (1996). Inferring complex phylogenies. Nature.

[b18] Ho SYW, Phillips MJ, Cooper A, Drummond AJ (2005). Time dependency of molecular rate estimates and systematic overestimation of recent divergence times. Mol. Biol. Evol.

[b19] Ho SY, Lanfear R, Bromham L, Phillips MJ, Soubrier J, Rodrigo AG (2011). Time-dependent rates of molecular evolution. Mol. Ecol.

[b20] Katoh K, Toh H (2010). Parallelization of the MAFFT multiple sequence alignment program. Bioinformatics.

[b21] Kishino H, Hasegawa H (1989). Evaluation of the maximum likelihood estimate of the evolutionary tree topologies from DNA sequence data, and the branching order in Hominoidea. J. Mol. Evol.

[b22] Lutjeharms JRE (2006). Three decades of research on the greater Agulhas Current. Ocean Sci Discuss.

[b23] Murphy WJ, Eizirik E, Johnson WE, Zhang YP, Ryder OA, O'Brien SJ (2001). Molecular phylogenetics and the origins of placental mammals. Nature.

[b24] Poe S, Swofford DL (1999). Taxon sampling revisited. Nature.

[b25] Posada D, Crandall ED (1998). Modeltest: testing the model of DNA substitution. Bioinformatics.

[b26] Rahbek C (2005). The role of spatial scale and the perception of large-scale species-richness patterns. Ecol. Lett.

[b200] Rambaut A, Suchard MA, Xie D, Drummond AJ (2013). http://beast.bio.ed.ac.uk/Tracer.

[b27] Ridgway TM, Stewart BA, Branch GM, Hodgson AN (2006). Morphological and genetic differentiation of *Patella granularis* (Gastropoda: Patellidae): recognition of two sibling species along the coast of Southern Africa. J. Zool.

[b28] Robba E (1987).

[b29] Ronquist F, Teslenko M, van der Mark P, Ayres DL, Darling A, Hohna S (2012). MrBayes 3.2: efficient Bayesian phylogenetic inference and model choice across a large model space. Syst. Biol.

[b30] Shimodaira H (2002). An approximately unbiased test of phylogenetic tree selection. Syst. Biol.

[b31] Shimodaira H, Hasegawa H (1999). Multiple comparisons of log-likelihoods with applications to phylogenetic inference. Mol. Biol. Evol.

[b32] Shimodaira H, Hasegawa H (2001). CONSEL: for assessing the confidence of phylogenetic tree selection. Bioinformatics.

[b33] Silva EC, Barros F (2011).

[b34] Sonnenfeld P, Stanley DJ, Wezel FC (1985). Models of upper Miocene evaporite genesis in the Mediterranean region. Geological evolution of the mediterranean basin.

[b35] Souza RCCLd, Fernandes FC, Lima TA, Silva EP (2008).

[b36] Stephenson TA, Stephenson A (1972). Life between tidemarks on rocky shores.

[b37] Stilwell JD, Zinsmeister WJ (1992). Molluscan systematics and biostratigraphy: lower Tertiary, La Meseta formation, Seymour Island.

[b38] Teske PR, McQuaid CD, Froneman PW, Barker NP (2006). Impacts of marine biogeographic boundaries on phylogeographic patterns of three South African estuarine crustaceans. Mar. Ecol. Prog. Ser.

[b39] Teske PR, Froneman PW, McQuaid CD, Barker NP (2007). Phylogeographic structure of the caridean shrimp Palaemon peringueyi in South Africa: further evidence for intraspecific genetic units associated with marine biogeographic provinces. Afr. J. Mar. Sci.

[b40] Teske P, Papadopoulos I, Newman B, Dworschak P, McQuaid C, Barker N (2008). Oceanic dispersal barriers, adaptation and larval retention: an interdisciplinary assessment of potential factors maintaining a phylogeographic break between sister lineages of an African prawn. BMC Evol. Biol.

[b41] Tuinen MV, Sibley CG, Hedges SB (2000). The early history of modern birds inferred from DNA sequences of nuclear and mitochondrial ribosomal genes. Mol. Biol. Evol.

[b42] Wagstaff SJ, Garnock-Jones PJ (1998). Evolution and biogeography of the Hebe complex (Scrophulariaceae) inferred from ITS sequences. New Zeal. J. Botany.

[b43] White TJ, Bruns T, Lee S, Innis MA, Gelfand DH, Sninsky JJ, White TJ, Taylor J (1990). Amplification and direct sequencing of fungal ribosomal RNA genes for phylogenetics. PCR Protocols: a guide to methods and applications.

[b44] Wiens JA (1989). Spatial scaling in ecology. Funct. Ecol.

[b45] Wood AR, Apte S, MacAvoy ES, Gardner J (2007). A molecular phylogeny of the marine mussel genus *Perna* (Bivalvia: Mytilidae) based on nuclear (ITS1&2) and mitochondrial (COI) DNA sequences. Mol. Phylogenet. Evol.

[b46] Yang Z (2007). PAML 4: a program package for phylogenetic analysis by maximum likelihood. Mol. Biol. Evol.

[b47] Zardi GI, McQuaid CD, Teske PR, Barker NP (2007). Unexpected genetic structure of mussel populations in South Africa: indigenous *Perna perna* and invasive *Mytilus galloprovincialis*. Mar. Ecol. Prog. Ser.

[b48] Zardi G, Nicastro K, McQuaid C, Hancke L, Helmuth B (2011). The combination of selection and dispersal helps explain genetic structure in intertidal mussels. Oecologia.

[b49] Zwickl DJ, Hillis DM (2002). Increased taxon sampling greatly reduces phylogenetic error. Syst. Biol.

