# Supplementary figures and images for: Wider sampling reveals a non-sister relationship for geographically contiguous lineages of a marine mussel
Source: Ecol Evol. 2014 Apr 25;4(11):2070–81. doi: 10.1002/ece3.1033 (PMC4201422; doi:10.1002/ece3.1033)

ML COI tree -ln L = 4400.22  
623 bp, 329 parsimony-informative

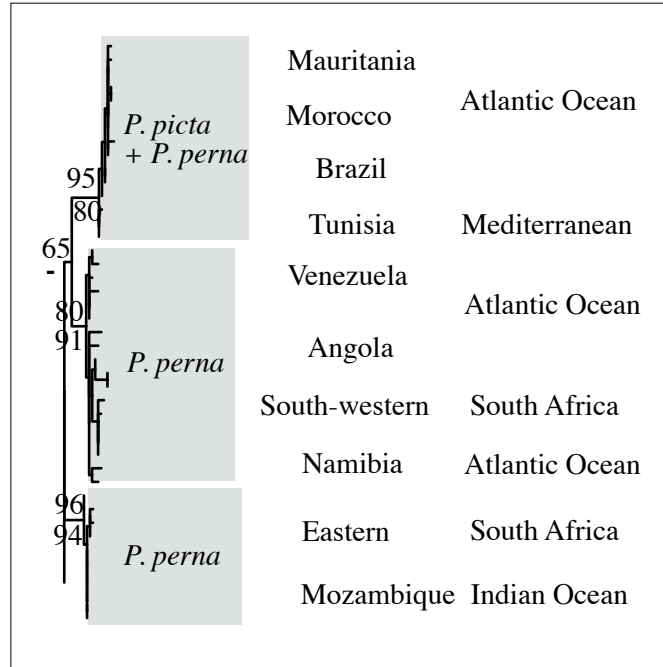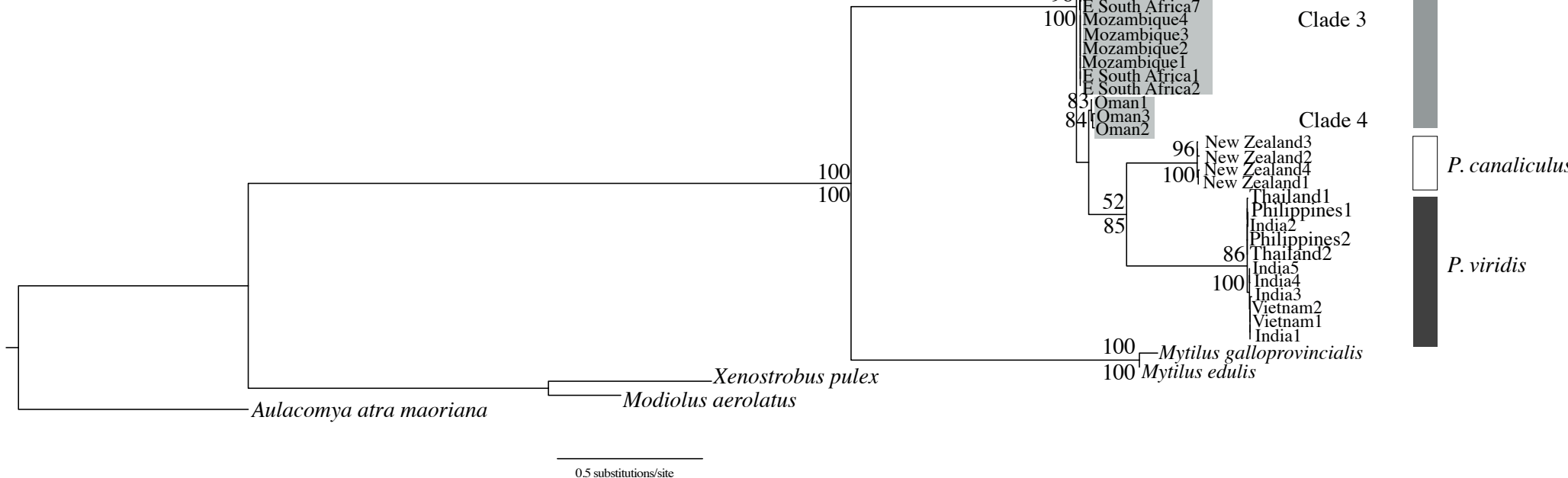

Supplement: Supplementary file 1 — Material S1. Phylogenetic relationships of the currently recognized species within the genus Perna. The maximum likelihood topology of a mitochondrial data set (COI) is shown. Numbers above and below nodes correspond to ML bootstrap values and Bayesian posterior probabilities, respectively. Gray boxes indicate the three clades within P. perna recovered in this analysis. The inset shows phylogeographic patterns of diversification within P. perna. [file ece30004-2070-sd1.pdf]

ML ITS tree -ln L = 2957.27  
865 bp

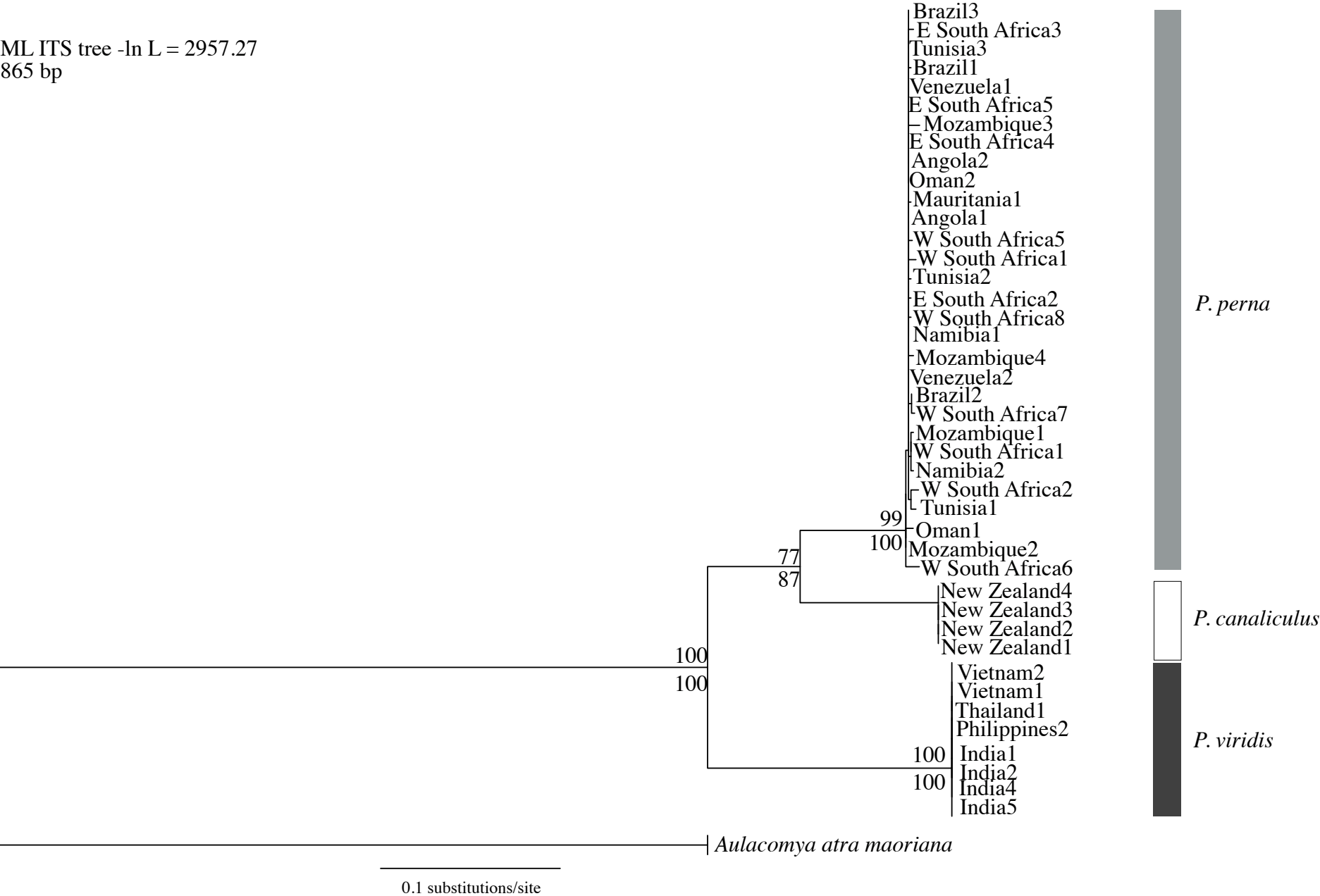

Supplement: Supplementary file 2 — Material S2. Phylogenetic relationships of the currently recognized species within the genus Perna. The maximum likelihood topology of a nuclear data set (ITS) using the GTR+Γ evolutionary model is shown. Numbers above and below nodes correspond to ML bootstrap values and Bayesian posterior probabilities, respectively. [file ece30004-2070-sd2.pdf]
